# Coupling the normal incident light into waveguide modes of DBR mirrors via a diffraction grating

**DOI:** 10.1038/srep38964

**Published:** 2016-12-13

**Authors:** Wenhong Yang, Shang Sun, Chen Zhang, Jiankai Li, Zonghui Duan, Qinghai Song, Shumin Xiao

**Affiliations:** 1 Department of Material Science and Engineering, Harbin Institute of Technology, Shenzhen, 518055, China; 2 National Key Laboratory on Tunable Laser Technology, Department of Electrical and Information Engineering, Harbin Institute of Technology, Shenzhen, 518055, China

## Abstract

Here we numerically and experimentally demonstrate the conversion of normally incident light into the guiding modes of distributed Bragg reflector (DBRs) mirror. By fabricating a gold grating onto a 7.5 pairs TiO_2_/SiO_2_ DBR mirror, a series of asymmetrical resonances have been formed at the bandgap range of the DBR mirror. The detailed numerical calculations show that these Fano resonances are attributed to the coupling of incident waves into guiding modes of the DBR mirror. Compared with the other resonances, this coupling mechanism can be simply realized and it has also been revealed to be quite robust to the environmental changes, making the conversion between propagating waves and guiding waves to be practically interesting for many applications.

It has been well recognized that a photonic band gap (PBG) could be generated by the interference of Bragg scattering in a periodic dielectric structures^1^. The simplest examples are the one-dimensional photonic crystals^2^, which are formed by stacking periodic pairs of thin films with different refractive indices. In past decades, the TiO_2_/SiO_2_^3^, AlN/GaN^4^, and porous silicon^5^ based one-dimensional photonic crystals have been realized and a number of practical applications have been developed, e.g. band-pass filters, beam splitters, and polarizers. By introducing the defect layers, one-dimensional photonic crystals have also been utilized to control the spontaneous emission^3^ and the light-matter strong coupling^6^,^7^,^8^. In additional to the PBG, the one-dimensional photonics crystals can also support the in-plane guiding modes. Such kind of guiding modes can trap the light well to form a new type of waveguide and has triggered many novel applications such as the generation of entangled photon pairs^9^,^10^. Once the material absorption has been considered, the in-plane guiding modes can overcome the limitation of extremely short absorption length (thickness) and thus significantly improve the performances of photovoltaic devices. While the guiding modes have so many advantages, they are actually quite difficult to be directly excited in experiments. Several groups have successfully coupled light into the guiding modes via a prism coupler^11^, which is too complicated and thus hinders the practical applications. Herein, by using a Au grating, we experimentally and numerically demonstrate that the normally incident light can be simply converted into the guiding modes of one-dimensional photonic crystals.

## Results

**Figure 1:**
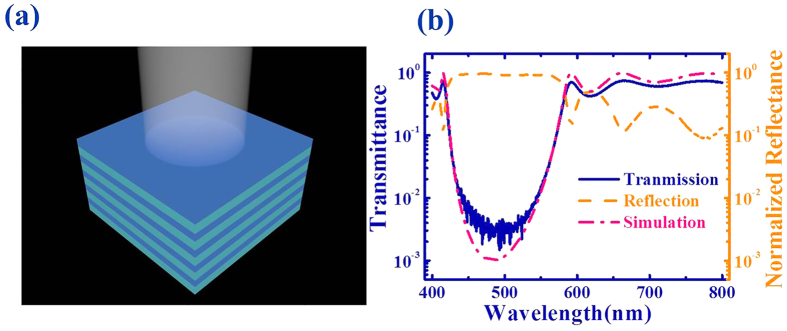
(**a**) The schematic picture of the DBR mirror. (**b**) The transmission (solid line) and reflection (dashed line) spectra of DBR mirror with normal incident. The dash-dotted line is the simulated transmission spectrum.

Figure 1(a) shows the schematic picture of the DBR mirror. It consists of 7.5 pairs of TiO_2_/SiO_2_ thin films on a glass substrate. In this research, the thicknesses of TiO_2_ and SiO_2_ thin films were 52 nm and 80 nm. And the refractive indices were around 2.41 and 1.47 at the designed wavelength (~500 nm)^12^, respectively. The fabrication detailed can be found in Methods. Then the transmission and reflection spectra have been recorded by using a home-made optical setup. The results are shown in Fig. 1(b), where a clear transmission gap can be found within the wavelength range from 420 nm to 560 nm. By using a transfer matrix^3^, the transmission spectrum has also been simulated. As the dashed line in Fig. 1(b), it matched to the experimental results very well. From the simulation, we know that the transmission gap was the conventional PBG and it could be simply explained by the interference between the reflected waves from the series interface between TiO_2_ and SiO_2_.

**Figure 2:**
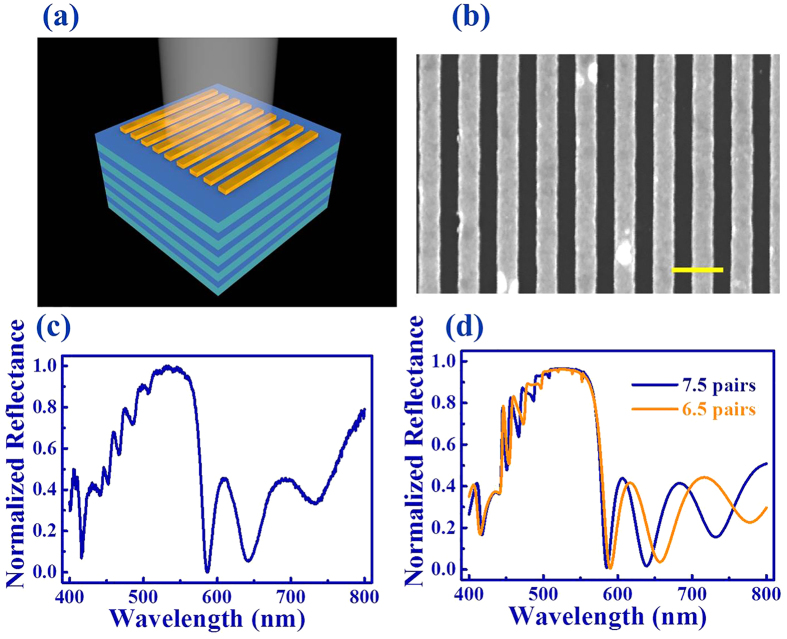
(**a**) The schematic picture of the DBR mirror with a Au grating. (**b**) The top-view SEM image of the grating. The scale bar is 400 nm. (**c**) The normalized reflection spectrum of TE modes of the DBR mirror with a grating. (**d**) The numerically calculated reflection spectrum with 7.5 pairs DBR (blue) and 6.5 pairs DBR (orange), respectively.

The situation drastically changed after Au nanostructures had been fabricated on the top of DBR mirror (see the schematic picture in Fig. 2(a)). The Au nanostructures were fabricated with electron beam lithography technique followed by liftoff process. In general, a 300 nm PMMA film (A4, Microchem) was spin-coated onto the DBR mirror and baked at 160 °C for an hour. As the substrate and the DBR mirror were both insulators, the sample was coated with 10 nm Al film and exposed to electron beam in E-beam writer (Raith E-line, 20 kV). The grating structure was obtained by removing Al film with HCl (18.5%) and developing the PMMA in MIBK/IPA (1:3) solution for 40 seconds and rinsed in IPA for 20 seconds. After that, the sample was placed into the E-beam evaporator and directly coated with 35 nm gold films (deposition rate 0.1 A/s, base vacuum pressure 5 × 10^−7^ torr). By immersing the sample in remover PG for 8 hours, the PMMA was removed and the grating was transferred to a reverse Au grating. Figure 2(b) shows the top-view scanning electron microscope image of the Au grating. Similar to the design, the width of Au ridge was 150 nm and the period of Au grating was 292 nm.

Then the optical properties of DBR mirror with grating have also been examined. The recorded reflection spectrum is shown in Fig. 2(c). Here the incident light was transverse electric (TE, with electric field along the directions of Au strips) polarized. While a broad reflection band at 420 nm–560 nm were very similar to Fig. 1(b), a number of resonant dips have been clearly observed at 524.5 nm, 507.9 nm, 487.0 nm, 467.5 nm, 452.3 nm, and 442.2 nm. The corresponding full width at half maximum (FWHMs) were 5.1 nm, 5.0 nm, 6.0 nm, 5.4 nm, 6.4 nm and 4.4 nm, respectively. The highest quality (Q) factor was even around 102. These dips are intrinsically different from the bare DBR mirror and thus it is interesting to reveal the underlying mechanisms. Intuitively, similar dips have been observed and were considered as the Fabry-Perot resonances, which formed by the interference between the reflections from the grating and the DBR mirror. This kind of resonances has been studied before to achieve surface emitting lasers^13^,^14^. However, these Fabry-Perot resonances usually require a defect gap between grating and DBR mirror. This gap, however, is absent in our study. And the reflection of gold grating was only around 30%, which is too low to form high Q resonances. Moreover, the Fabry-Perot resonances can also be excluded by the resonant wavelengths. In Fig. 2(c), the resonant frequencies are quit random and far away from equal spacing of conventional Fabry-Perot resonances.

**Figure 3:**
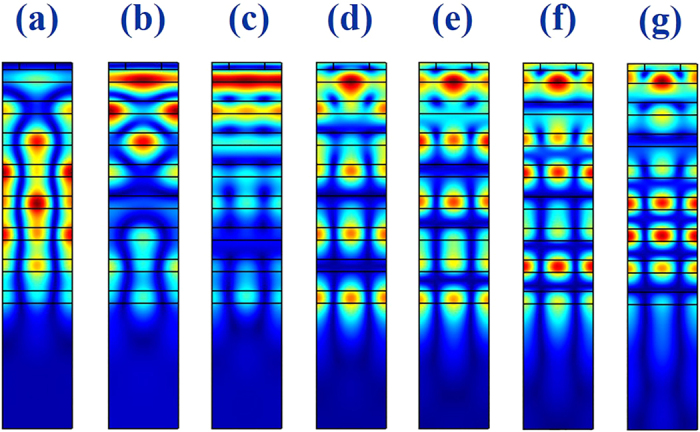
The numerically calculated field patterns of TE polarized waves at 553 nm, 542 nm, 527 nm, 508 nm, 487 nm, 467 nm, and 451 nm, respectively. The modes are the fundamental waveguide mode to 6^th^ order waveguide mode, respectively.

To well understand these resonances, we have numerically studied the experimental structures with a finite element method based software (Comsol multiphysics)^15^. The refractive indices of TiO_2_, SiO_2_, and the permittivity of gold were taken from the experimental results^12^. Figure 2(d) shows the numerically calculated results with TE polarization. We can also see the resonant dips at the positons around 527 nm, 508 nm, 487 nm, 467 nm, and 451 nm. All these resonant wavelengths match the experimental results well. Figure 3(a–g) shows the field patterns of dips at 553 nm, 542 nm, 527 nm, 508 nm, 487 nm, 467 nm, and 451 nm, respectively. We can see that the incident waves are well localized within the DBR mirror. Interestingly, the fields out of the DBR mirror show obviously evanescent decays, clearly indicating the confinements along the guiding modes. The different dips in the spectrum just related to the different order of guiding modes, which is confirmed by the field pattern shown in Fig. 3(a–g). While the field distributions are segmented by the multilayers, the maximal field can still be categorized into 1–7 groups in Fig. 3(a–g). Consequently, these modes correspond to the fundamental to 6^th^ order waveguide modes inside the multilayers.

In order to further distinguish the Fabry-Perot resonances and guiding modes, we have also calculated the reflection spectrum by changing the DBR from 7.5 pairs to 6.5 pairs. For the Fabry-Perot resonances, this changing can only form the changes in FWHMs, which are dependent on the reflectance of DBR mirror. In case of guiding modes, the waveguide modes are dependent on the waveguide total thickness. Thus the dip positions shall be changed when the total width of waveguide was changed. The results are also plotted in Fig. 2(d). From the changes in resonant wavelengths, we can also know that the resonances are formed by the transition between propagating waves into guiding modes.

**Figure 4:**
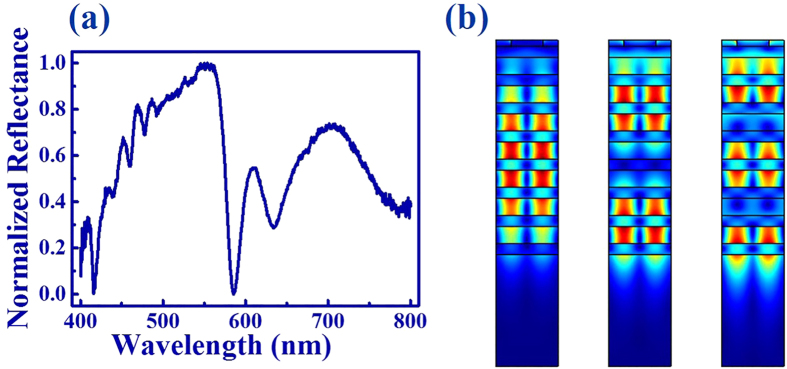
(**a**) The normalized reflection spectrum of TM polarization. (**b**) The corresponding numerically calculated field patterns of resonances at 501 nm (left), 491 nm (middle), and 476 nm (right).

**Figure 5:**
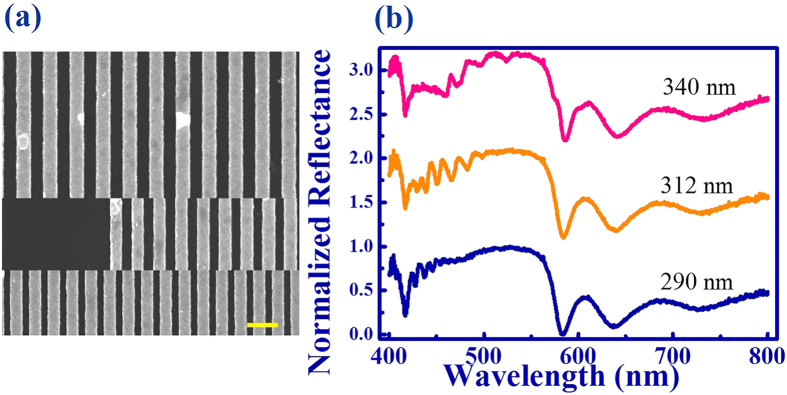
(**a**) Top, middle, and bottom panels are the top-view SEM images of the grating with period 340 nm, 312 nm, and 290 nm. The scale bar is 400 nm. (**b**) The dependence of normalized reflection spectra with different grating period.

In additional to the TE polarization, similar phenomena also hold true for the transverse magnetic (TM, with E perpendicular to the ridges) polarized light. As shown in Fig. 4(a), a number of resonant dips have also been observed at around 492.0 nm, 477.3 nm, 459.2 nm, and 440.7 nm, respectively. Due to the symmetry of Au grating, these resonant dips were different from the ones of TE polarization (see Fig. 2(c)). The TM polarized reflection has also been numerically studied. Similar to the TE polarization, all the resonant dips match the experimental results well. Figure 4(b) show the field patterns of the TM polarized modes at 501 nm (left), 491 nm (middle), and 476 nm (right). They were also the fundamental, first order, and second order TM waveguide modes, respectively. Figure 5(b) shows the TE polarized reflection spectra of the same DBR mirror with different grating period (see the SEM images in Fig. 5(a)). With the period of grating changed from 340 nm to 290 nm (the width of Au strip was always half of the period), there were always resonant dips could be clearly observed. In this sense, we know that the normally incident propagating waves could be simply converted to guiding modes by applying a grating on top of the DBR mirror, with no dependent on the width of grating and polarizations of incident wave.

**Figure 6:**
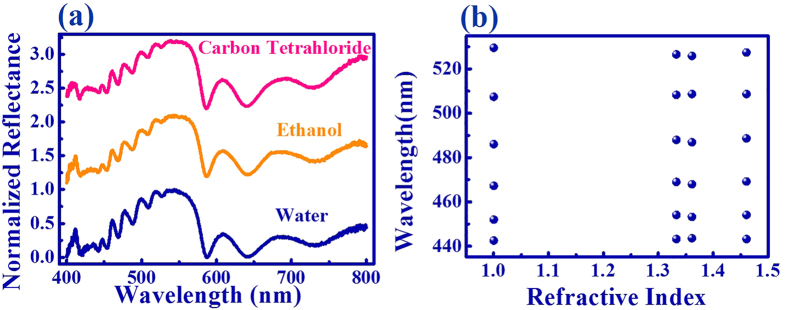
The wavelengths of resonant dips as a function of environmental refractive index.

For most of the resonant devices, the operating wavelengths are usually dependent on the environmental refractive index^16^,^17^,^18^. While it can be applied as environmental or biological sensor, these devices are quite difficult to be practically applied. Here we note that the wave conversion in this study is quite robust. As shown in Fig. 6, even though the environmental materials have been changed from air (see Fig. 2(c)), water, ethanol, to carbon tetrachloride, the resonant dips were well kept at the same positions, making the developed mechanism to be suitable for various conditions. This kind of robustness is also consistent with the field distributions. As shown in Fig. 4, the incident waves are confined within the waveguide modes and far away from the top surface. In this sense, the resonant positions are independent on the coating materials and are less affected by the environmental changes.

## Discussion

In summary, we have studied a simple method to convert propagating waves into guiding waves of DBR mirrors. This coupling mechanism was found to be suitable for both TE and TM polarization and was very robust to the environmental changes. This method has the potential to significantly enhance the light absorption lengths of photovoltaic thin films. In addition, this method is not limited in the DBR mirrors, it can also be used to couple light into the guiding modes within the defect layers of photonic crystals.

## Methods

The TiO_2_ and SiO_2_ thin films were deposited on glass substrate by electron beam (E-beam) evaporator (Syskey Co., Ltd)^12^. The deposition rates of TiO_2_ and SiO_2_ films were fixed at 1 angstrom/s and 5 angstrom/s, respectively. The electron gun operating voltage was 5.2 kv, and the current of beam was 4.5 mA. The vacuum of deposition was below 5.0 × 10^−7^ torr. The substrate temperature during deposition was kept at 200 °С. The refractive indices of TiO_2_ and SiO_2_ thin films were measured by ellipsometry.

## Additional Information

**How to cite this article:** Yang, W. *et al*. Coupling the normal incident light into waveguide modes of DBR mirrors via a diffraction grating. *Sci. Rep.* **6**, 38964; doi: 10.1038/srep38964 (2016).

**Publisher’s note:** Springer Nature remains neutral with regard to jurisdictional claims in published maps and institutional affiliations.
